# Medical and Surgical History of the Rebellion

**Published:** 1862-08

**Authors:** William A. Hammond

**Affiliations:** Surgeon General, U. S. A.


MEDICAL AND SURGICAL HISTORY OF THE REBELLION.—It is intended to prepare for publication the Medical and Surgical History of the Rebellion.

The medical portion of this work has been committed to Assistant Surgeon J. J. Woodward, United States Army, and the surgical part to Brigade Surgeon John H. Brinton, United States Volunteers.

All medical officers are, therefore, requested to co-operate in this undertaking by forwarding to this office such sanitary, topographical, medical and surgical reports, details of cases, essays, and results of investigations and inquiries, as may be of value for this, for which full credit will be given in the forthcoming volumes.

It is, therefore, confidently expected that no one will neglect this opportunity of advancing the honor of the service, the cause of humanity, and his own reputation,

WILLIAM A. HAMMOND,
Surgeon General, U. S. A.

